# The relationship between cardiometabolic disease and vertebral bone strength, mineral density, and mass in US adults

**DOI:** 10.1210/jendso/bvag193

**Published:** 2026-10-26

**Authors:** Nicole C Wright, Rafeka Hossain, Mary L Bouxsein, Tony M Keaveny, David C Lee, Gerhard Hellemann, John Jeffrey Carr, James G Terry, Amy H Warriner, Cora E Lewis

**Affiliations:** Center for Health Outcomes, Implementation, and Community-Engaged Sciences, Tulane University, New Orleans, LA 70112, USA; Center for Health Outcomes, Implementation, and Community-Engaged Sciences, Tulane University, New Orleans, LA 70112, USA; Center for Advanced Orthopaedic Studies, Beth Israel Deaconess Medical Center, Harvard University, Boston, MA 02215, USA; O.N. Diagnostics, Berkeley, CA 94704, USA; O.N. Diagnostics, Berkeley, CA 94704, USA; Department of Biostatistics, School of Public Health, University of Alabama at Birmingham, Birmingham, AL 35233, USA; Department of Radiology, Vanderbilt University Medical Center, Nashville, TN 37232, USA; Department of Radiology, Vanderbilt University Medical Center, Nashville, TN 37232, USA; Division of Endocrinology, Diabetes, and Metabolism, School of Medicine, University of Alabama at Birmingham, Birmingham, AL 35233, USA; Department of Epidemiology, School of Public Health, University of Alabama at Birmingham, Birmingham, AL 35233, USA

**Keywords:** cardiometabolic disease, bone strength, biomechanical computed tomography, epidemiology

## Abstract

**Context:**

Cardiometabolic disease (CMD) may increase vertebral fracture risk through deterioration in bone structure and/or tissue properties, compromising bone strength.

**Objective:**

Evaluate the relationship between CMD and vertebral bone measures.

**Design:**

Cross-sectional.

**Participants:**

Coronary Artery Risk Development in Young Adults (CARDIA) study participants with year 25 (2010-2011) abdominal computed tomography (CT) scans (n = 2728).

**Exposure:**

CMD (abdominal obesity, diabetes mellitus, dyslipidemia, or hypertension) status (≥1 vs 0) and burden (0, 1, 2, ≥3).

**Outcome Measure(s):**

Vertebral strength, volumetric bone mineral density (vBMD), and bone mass, obtained via biomechanical CT analysis.

**Methods:**

Multivariable linear regression evaluated the association between CMD and vertebral bone measures. Sex-specific models were adjusted for age, race, height, weight, and additional social determinants of health, lifestyle factors, and comorbidities. Effect modification by race, age, and menopausal status was evaluated.

**Results:**

Overall, at least 1 CMD was identified in 72.3% of men and 70.5% of women. In the fully adjusted model, CMD status was significantly associated with lower total (β = −6.32 mg/cm^3^; *P* = .018), trabecular (β = −4.53 mg/cm^3^; *P* = .048), and cortical (β = −6.63 mg/cm^3^; *P* = .034) vBMD in men. Greater CMD burden was associated with lower total and trabecular vBMD. The negative association between CMD and vBMD was stronger in men ≤50 years. There was no association in women.

**Conclusion:**

In this biracial cohort of Black and White adults from the United States, CMD was associated with significantly lower total and subcompartment vertebral vBMD in men.

Cardiometabolic diseases (CMDs) are an interconnected group of conditions with shared pathophysiology, including obesity, insulin resistance, dyslipidemia, hypertension (HTN), and inflammation (1). CMDs account for ∼20 million deaths globally (2). In addition to mortality, CMD increases the risk for other health conditions, including osteoporotic fractures.

Osteoporotic fractures are highly prevalent (3-5), have a significant economic burden (3, 6, 7), and are associated with many adverse outcomes, including death (8, 9). Vertebral fractures are the most common osteoporotic fractures (10). Regarding CMD and vertebral fractures, the most is known about type 2 diabetes mellitus (T2DM). Individual studies and meta-analyses have highlighted that T2DM increases the risk for vertebral fractures by ∼40% (11-16). Abdominal obesity has been shown to be associated with an 18% higher risk of vertebral fractures in a meta-analysis (17), with more recent studies reporting a higher risk of vertebral fractures in those with abdominal obesity based on waist circumference (18) even in those with lower body fat percentage (19) or those with normal body mass index (20). The relationship between dyslipidemia and vertebral fractures is mixed, with studies showing lower triglycerides in those with incident fractures (21, 22), while others have shown higher triglycerides and high-density lipoprotein cholesterol (HDL-C) were associated with vertebral fractures (23, 24). Similarly, studies specifically evaluating HTN and vertebral fracture are mixed, with a study of Japanese postmenopausal women reporting HTN being associated with a 76% increased risk (25), whereas a Danish study reported HTN was associated with vertebral fracture only in those with a shorter duration of HTN (26). Lastly, medications used in the treatment of CMDs, such as thiazolidinediones (27), insulin (28, 29), sodium-glucose cotransporter 2 inhibitors (particularly canagliflozin) (30, 31), diuretics (both thiazide and loop), and calcium channel blockers (32), are associated with increased risk of vertebral fractures. Though studies have shown a relationship between the individual CMDs and fracture, many previous studies did not account for the CMD clustering effect, which is particularly important given the high multimorbidity within these conditions. Such clustering could have an additive or multiplicative effect on the skeleton.

Bone mineral density (BMD), measured via dual energy X-ray absorptiometry (DXA), is 1 of the strongest risk factors for fracture. However, paradoxically, BMD has been shown to be higher in those with CMDs (33), particularly T2DM and abdominal obesity. This suggests that 1 mechanism through which CMDs are associated with higher fracture risk involves deterioration in bone structure and/or bone tissue properties, compromising bone strength. Unfortunately, DXA, the gold standard for assessing BMD, does not directly measure bone strength.

Advanced analysis of clinical computed tomography (CT) scans, coined biomechanical computed tomography (BCT), uses finite element analysis to estimate bone strength from the measured volumetric BMD (vBMD) and vertebral geometry (34). Studies have shown that BCT-based vertebral strength predicts fracture better than or as well as DXA-BMD (35-39). However, to our knowledge, the potential relationship between CMD and BCT bone strength measures has not been studied. Given that recent data show that only 6.8% of adults in the United States (US) have optimal cardiometabolic markers (eg, adiposity and glucose) (40), and the projected increase in the prevalence of CMDs (41, 42), studying the relationship between CMD and vertebral strength measures will provide novel information on how these conditions as a collective could impact fracture risk.

## Methods

### Study design and setting

We conducted a cross-sectional study using data from the Coronary Artery Risk Development in Young Adults (CARDIA) study. CARDIA is a biracial, community-based cohort of Black and White US adults, established in 1985-1986 to examine cardiovascular disease development from young adulthood (ages 18-30 years at baseline) through midlife (43, 44). CARDIA participants were examined approximately every 5 years, completing a battery of questionnaires, providing blood specimens, and completing imaging, including chest and abdominal CT at some exams. The protocols were approved by Institutional Review Boards of the University of Alabama at Birmingham, Kaiser Permanente of Oakland, California; University of Minnesota; and Northwestern University. Signed informed consent was obtained from all participants at every examination at their respective field centers (Birmingham, AL; Chicago, IL; Minneapolis, MN; Oakland, CA).

This cross-sectional component of the CARDIA-Bone ancillary study, approved by the Tulane University Institutional Review Board, included 1000 men and 1777 women with year 25 (2010-2011) abdominal CT scans, encompassing a random selection of participants within each race and sex group (Fig. 1). Our sample had an equal distribution of Black and White participants within each sex group as in the parent study but included more women (∼2/3) than men by design. We excluded participants with an estimated glomerular filtration rate of <30 mL/min/1.73 m^2^, calculated using the Chronic Kidney Disease Epidemiology Collaboration equation without race, due to the possibility of chronic kidney disease–mineral bone disorder.

**Figure 1:**
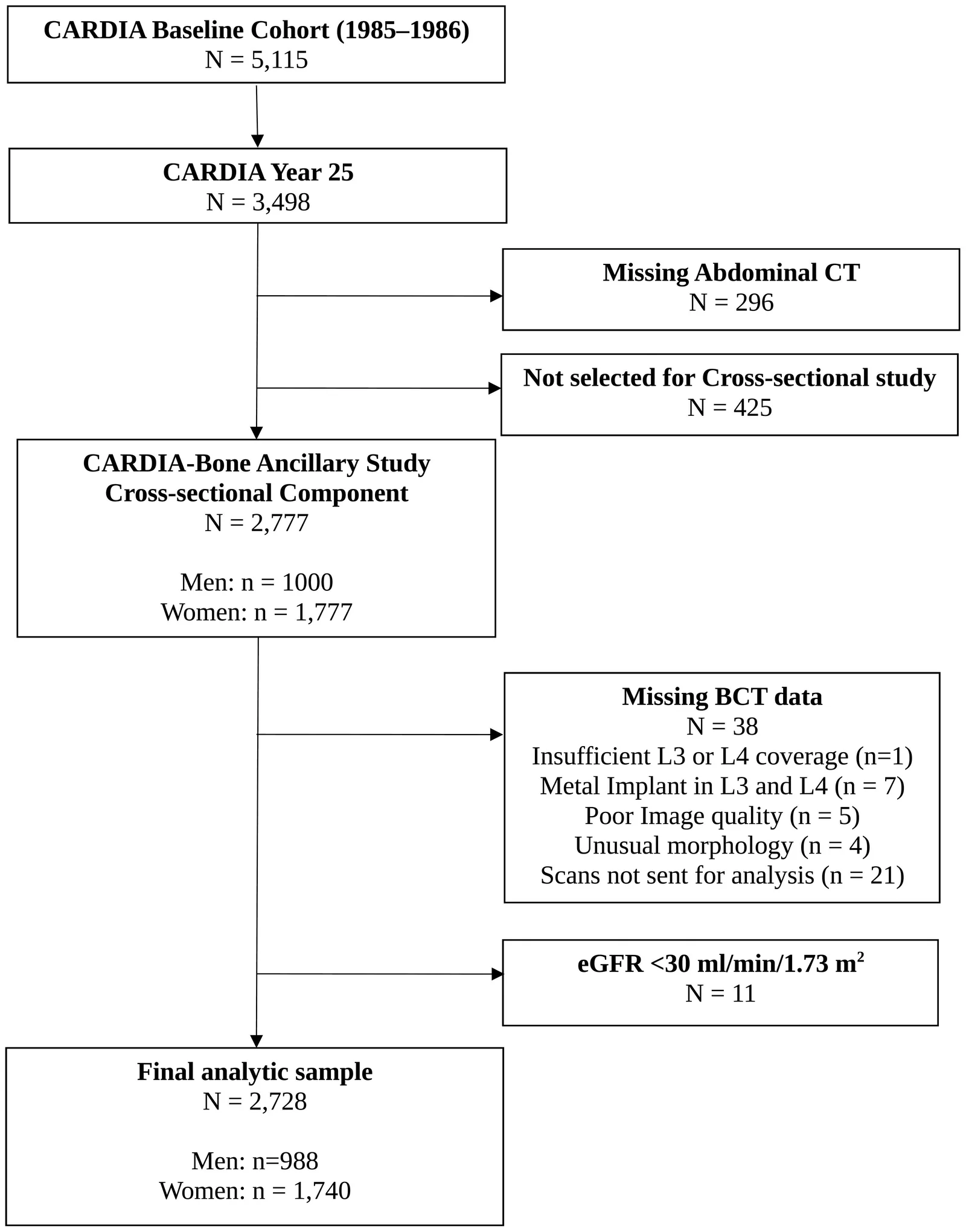
Participant selection flowchart.

### Exposure: cardiometabolic disease

Our CMD exposure included the following 4 conditions: abdominal obesity, diabetes mellitus (DM), dyslipidemia, and HTN. Abdominal obesity was based on waist circumference, measured via standard protocols at the year 25 visit, and categorized as a binary variable using the thresholds that define metabolic syndrome (≥102 cm in men; ≥88 cm in women) (45). DM was identified in those with a fasting plasma glucose level ≥126 mg/dL, 2-hour oral glucose tolerance test ≥200 mg/dL, HbA1c ≥6.5%, self-reported use of DM, or self-reported DM medications (46). Dyslipidemia was identified in participants with triglycerides ≥150 mg/dL, HDL-C <40 mg/dL, or self-reported use of lipid-lowering medications. Triglycerides and HDL-C were chosen over other lipid measures as they more quantitatively reflect metabolic dyslipidemia (47). Given that statins, the predominant lipid-lowering medications used, mostly target low-density lipoprotein cholesterol, we created a second dyslipidemia variable for a sensitivity analysis that excluded those meeting the dyslipidemia definition only via self-report of lipid-lowering medications. HTN was identified as a mean systolic blood pressure (BP) ≥130 mm Hg (48, 49) or mean diastolic BP ≥80 mm Hg (an average of the second and third BP measurement) (48, 49), or self-reported use of antihypertensive medications. We created 2 CMD exposure variables: (1) CMD status (presence of at least 1 CMD) as a binary variable and (2) CMD burden as a categorical variable (0, 1, 2, or ≥3).

### Outcome: BCT-based vertebral strength, mineral density, and size

The CARDIA abdominal CT scans were acquired using 64-channel multidetector GE (GE Healthcare Milwaukee, WI, USA; Birmingham and Oakland centers) and Siemens (Siemens, Erlangen, Germany; Chicago and Minneapolis centers) scanners (50). A standardized helical scan protocol limiting coverage to the lower lumbar vertebrae was used to capture images of L3 to L5. Technical parameters were standardized across the CT systems (120 kVp, 1-mm or 1.25-mm helical slices, 0.984 pitch, 0.5-second rotation, 35-cm display field of view with 50-cm reconstruction to provide full subcutaneous adipose coverage). Tube current was 320 mA for GE scanners and 180 mAs for Siemens scanners, and tube currents were increased by 25% for individuals weighing >100 kg (51, 52). Each participant was scanned lying on a quality control phantom with known quantities of calcium hydroxyapatite and fat (INTable Calibration Pad; Image Analysis, Inc., Columbia, KY, USA). Radiology technologists were trained and certified on the research protocol, and scans were centrally monitored in near real-time to ensure cross-site consistency. CT images were anonymized and electronically transmitted to the central CT reading center located at Wake Forest University School of Medicine (Winston-Salem, NC, USA).

BCT analysis using the VirtuOst software (O.N. Diagnostics, Berkeley, CA, USA) was performed at L3 or L4 when L3 was not usable (n = 88 [3.2%] of scans) on the de-identified abdominal CT scans. Details of BCT analysis have been previously published, but in summary, BCT combines finite element analysis and biomechanical modeling to perform a virtual stress test to compute a measurement of bone strength, which is the force required to virtually break or fracture the patient's spine in a standardized loading configuration (38, 39, 53-56). In addition to vertebral strength, other data obtained from BCT analysis included vBMD and measurements of vertebral size (mass and vertebral body cross-sectional area). The strength, density, and size parameters were generated for the total vertebral body as well as the trabecular and cortical compartments. The average coefficients of variation re-analysis precision (different analyst, same scan) for vertebral strength, mid-vertebral trabecular vBMD, and total vBMD are 0.5%, 0.5%, and 0.3%, respectively (57). The respective short-term precision values (same analyst, repeat scans of the same patient) is estimated to be 1.5%, 1.3%, and 0.7% (data on file at O.N. Diagnostics). Our vertebral BCT outcomes included total vertebral strength (newtons), total vBMD (mg/cm^3^), trabecular (mid-vertebral) vBMD, cortical vBMD, and total mass (g).

### Covariates

We evaluated the following variables as potential confounders in the relationship between CMD and vertebral strength: sociodemographic (age and race), anthropometric (height and weight), social determinants of health (education level, family income, employment status), nutritional (calcium and vitamin D intake), lifestyle (smoking status, alcohol consumption, physical activity), other comorbidities that could be related to CMD or bone health (thyroid disease, digestive disease, liver disease, depression, multiple sclerosis, chronic obstructive pulmonary disease, cancer), and antidepressants and corticosteroid medications. In women, we also included menopausal status, polycystic ovary syndrome, and use of menopausal hormone therapy. Except for height and weight, which were assessed using standardized protocols at clinic visits, all covariate data were self-reported. Calcium and vitamin D intake data were only assessed in year 20 using the CARDIA diet history (58). Given habitual food choices in adults (59), we carried forward the year 20 estimates for these analyses. Alcohol consumption was summarized as the number of drinks (wine, beer, or liquor) per week. Physical activity was calculated as total intensity score in exercise units, with 300 exercise units approximately equivalent to 150 minutes per week of moderate-to-vigorous physical activity (60). Corticosteroids included both oral and inhaled medications.

### Statistical analysis

We described the sample characteristics overall and by CMD status (≥1 CMD variable). Given known differences in BMD by sex (61, 62), all analyses were stratified by sex. Differences in characteristics were identified using *t* tests for continuous variables and χ² tests for categorical variables. For each of the 2 exposure variables, multivariable linear regression models were used to estimate associations between CMD and each vertebral BCT outcome variable. We first adjusted for age, race, height, and weight given the high correlation of the BCT variables with age and body size (63, 64). The fully adjusted model included significant social determinants of health, nutritional, lifestyle, comorbidities, and medication variables. For the categorical CMD burden variable, we also examined dose–response relationships using linear trend tests. Lastly, we evaluated whether race, age (≤50 vs >50 years), and menopausal status and hormone therapy use in women modified the association between CMD and the vertebral BCT variables using interaction terms (eg, CMD × Race). We stratified the results for all significant interaction terms (*P* < .05). All analyses were conducted in Stata v18.5 (StataCorp, College Station, TX, USA).

## Results

### Characteristics of the sample

The analytic sample comprised 2728 participants (Fig. 1), with ages ranging from 42 to 59 years; 63.8% were women, and 50.4% were Black. At least 1 CMD was identified in 714 (72.3%) of men and 1226 (70.5%) of women (Table 1). Men were more likely than women to have dyslipidemia (44.8% vs 26.9%), whereas women were more likely to have abdominal obesity than men (53.5% vs 37.7%). The CMD burden was substantial, with 31.0% of men and 29.5% of women having ≥3 CMDs. Men with at least 1 CMD were more likely to be older, have a higher mean weight, have lower physical activity scores, and be more likely to report cancer and antidepressant use. Women with at least 1 CMD were more likely to be Black, have a higher mean weight, have lower education and household income, have lower calcium and vitamin D intake, drink fewer alcoholic drinks per week, have lower physical activity scores, be current smokers, and greater report chronic obstructive pulmonary disease and antidepressant use.

**Table 1:** Characteristics of CARDIA participants by cardiometabolic disease status

	Men (n = 988)			Women (n = 1740)		
	CMD yes (n = 714)	CMD no (n = 274)	*P* value*^a^*	CMD yes (n = 1226)	CMD no (n = 514)	*P* value*^a^*
Sociodemographic						
Age, years, mean (SD)	50.2 (3.7)	49.6 (3.7)	.**022**	50.2 (3.7)	50.1 (3.7)	.436
Race, n (%)			.074			**<**.**001**
Black	371 (52.0%)	125 (45.6%)		746 (60.8%)	134 (26.1%)	
White	343 (48.0%)	149 (54.4%)		480 (39.2%)	380 (73.9%)	
Height, mean (SD)	177.9 (6.8)	177.5 (7.0)	.408	164.3 (6.8)	165.0 (6.3)	.053
Weight, mean (SD)	217.8 (44.5)	178.3 (24.7)	**<**.**001**	200.6 (47.4)	143.8 (20.6)	**<**.**001**
Social determinants of health, n (%)						
Education level			.458			**<**.**001**
≤High school	295 (41.3)	110 (40.1)		470 (38.3)	110 (21.4)	
Some college	133 (18.6)	41 (15.0)		270 (22.0)	74 (14.4)	
College graduate	159 (22.3)	70 (25.5)		296 (24.1)	183 (35.6)	
Postgraduate	126 (17.6)	51 (18.6)		186 (15.2)	145 (28.2)	
Family income			.823			**<**.**001**
<$35 000	184 (25.8)	65 (23.7)		363 (29.6)	79 (15.4)	
$35 000-<$50 000	62 (8.7)	25 (9.1)		156 (12.7)	49 (9.5)	
$50 000-<$75 000	104 (14.6)	45 (16.4)		232 (18.9)	96 (18.7)	
≥$75 000	359 (50.3)	133 (48.)		456 (37.2)	279 (54.3)	
Employment status			.247			.067
Employed for wages	562 (78.7)	230 (83.9)		918 (74.9)	412 (80.2)	
Homemaker	100 (14.0)	30 (10.9)		259 (21.1)	91 (17.7)	
Unemployed	42 (5.9)	11 (4.0)		35 (2.9)	8 (1.6)	
Nutritional, mean (SD)						
Calcium intake, mg	929.1 (747.6)	987.3 (859.8)	.294	870.2 (750.9)	1086.0 (867.0)	**<**.**001**
Vitamin D intake, mcg	7.9 (8.9)	7.8 (8.0)	.867	7.4 (10.9)	9.6 (16.9)	.**002**
Lifestyle						
Alcohol drinks/week, mean (SD)	7.2 (13.5)	7.4 (15.2)	.870	2.8 (5.7)	3.9 (5.5)	**<**.**001**
Smoking status, n (%)			.728			.**013**
Never	417 (58.4)	167 (60.9)		718 (58.6)	334 (65.0)	
Past	142 (19.9)	49 (17.9)		278 (22.7)	114 (22.2)	
Current	148 (20.7)	56 (20.4)		206 (16.8)	60 (11.7)	
Physical activity, mean (SD)						
Total intensity score, EU	377.7 (289.4)	443.7 (301.6)	.**002**	245.2 (226.3)	370.9 (265.5)	**<**.**001**
Comorbidities, n (%)						
Thyroid disease	18 (2.5)	7 (2.6)	.825	160 (13.1)	65 (12.6)	.933
Digestive disease	34 (4.8)	10 (3.6)	.719	106 (8.6)	36 (7.0)	.399
Liver disease	26 (3.6)	6 (2.2)	.286	40 (3.3)	11 (2.1)	.293
Depression	108 (15.1)	32 (11.7)	.205	343 (28.0)	133 (25.9)	.434
MS	0 (0.0)	0 (0.0)	—	7 (0.6)	3 (0.6)	.979
COPD	10 (1.4)	0 (0.0)	.080	24 (2.0)	2 (0.4)	.**046**
Cancer	40 (5.6)	6 (2.2)	.**033**	97 (7.9)	60 (11.7)	.**031**
Medications, n (%)						
Antidepressants	64 (9.0)	12 (4.4)	.**047**	210 (17.1)	64 (12.5)	.**019**
Corticosteroids	4 (0.6)	4 (1.5)	.158	29 (2.4)	8 (1.6)	.286
Women-specific reproductive factors, n (%)						
Postmenopausal				521 (42.5)	204 (39.7)	.279
Hormone therapy				96 (7.8)	45 (8.8)	.519
PCOS				53 (4.3)	14 (2.7)	.114
CMD details, n (%)						
Abdominal obesity	372 (37.7)			930 (53.5)		
Diabetes mellitus	149 (15.1)			301 (17.3)		
Dyslipidemia	443 (44.8)			467 (26.9)		
Hypertension	476 (48.2)			759 (43.7)		
CMD burden, n (%)						
1	279 (39.0)			465 (37.9)		
2	214 (30.0)			399 (30.6)		
3+	221 (31.0)			362 (29.5)		

Missing data: height: n = 2 (0.1%); weight: n = 3 (0.1%); education level: n = 9 (0.3%); family income: n = 41 (1.5%); alcohol consumption: n = 25 (0.9%); smoking status: n = 39 (1.4%); physical activity total intensity score: n = 23 (0.8%); thyroid disease: n = 9 (0.3%); digestive disease: n = 8 (0.3%); liver disease: n = 8 (0.3%); depression: n = 9 (0.3%); MS: n = 8 (0.3%); COPD: n = 8 (0.3%); cancer: n = 8 (0.3%); antidepressants: n = 12 (0.4%). Bold indicates *P* < .05.

Abbreviations: CMD, cardiometabolic disease (status assessed as a binary variable); COPD, chronic obstructive pulmonary disease; EU, exercise units; MS, multiple sclerosis; PCOS, polycystic ovarian syndrome.

^*a*^ *P* values for continuous variables are based on *t* test and χ² test for categorical variables.

### The association between CMD and vertebral BCT variables

#### Men

The mean total strength, total vBMD, and total mass did not differ by overall CMD status; however, men with at least 1 CMD had lower mean cortical vBMD than men without CMD (Mean [SD], 318 [40] vs 325 [40] mg/cm^3^; *P* = .016) (Table 2). In the crude regression analysis, CMD status was associated with significantly lower cortical vBMD (β, −6.92 mg/cm^3^; *P* = .016) (Table 3). After adjusting for age, race, height, and weight, CMD was marginally associated with lower total vertebral strength (β, −360.56 [186.74] newtons; *P* = .054), and significantly lower total, trabecular, and cortical vBMD (Table 3, Model 1) but was not associated with total mass (β, 0.16 [0.13] g; *P* = .215). In the fully adjusted model, CMD was associated with significantly lower total (β, −6.32 [2.68] mg/cm^3^; *P* = .018), trabecular (β, −4.53 [2.29] mg/cm^3^; *P* = .048), and cortical (β, −6.63 [3.12] mg/cm^3^; *P* = .034) vBMD (Table 3, Model 2). Similarly, there was a linear trend showing that greater CMD burden was associated with lower strength (Table 3, Model 1), and lower total and trabecular vBMD (Table 3, Model 2). Results were similar when dyslipidemia was defined based on lipid values alone compared with lipid values and self-reported medication use (data not shown).

**Table 2:** Mean (SD) vertebral bone strength, mineral density, and mass by cardiometabolic disease status

	CMD yes	CMD no	*P* value*^a^*
	Mean (SD)	Mean (SD)	
Men	n = 714	n = 274	
Total strength, newtons	11 320 (2621)	11 185 (2514)	.465
Total vBMD, mg/cm^3^	228 (38)	231 (38)	.379
Midvertebral vBMD, mg/cm^3^	145 (34)	145 (37)	.847
Cortical vBMD, mg/cm^3^	**318** (**40)**	**325** (**40)**	.**016**
Total mass, g	9.98 (1.86)	9.82 (1.84)	.215
Women	n = 1226	n = 514	
Total strength, newtons	9258 (2332)	9052 (2141)	.085
Total vBMD, mg/cm^3^	234 (43)	234 (40)	.968
Midvertebral vBMD, mg/cm^3^	**154** (**39)**	**147** (**37)**	**<**.**001**
Cortical vBMD, mg/cm^3^	**322** (**47)**	**334** (**43)**	**<**.**001**
Total mass, g	7.67 (1.53)	7.75 (1.42)	.312

Bold indicates *P* < .05.

Abbreviations: CMD, cardiometabolic disease (status assessed as binary variable); vBMD, volumetric bone mineral density.

^*a*^ *P* values from *t* tests comparing unadjusted means by overall CMD status.

**Table 3:** The association between cardiometabolic disease and vertebral bone strength, mineral density, and mass in men

	Crude		Model 1*^a^*		Model 2*^b^*	
	β (SE)	*P* value	β (SE)	*P* value	β (SE)	*P* value
Total strength, newtons						
CMD status, yes vs no	134.57 (184.19)	.465	−360.56 (186.74)	.054	−316.32 (190.00)	.096
CMD burden						
0	Ref.		Ref.		Ref.	
1	−0.09 (220.28)	1.000	−273.42 (204.65)	.182	−270.00 (208.27)	.195
2	31.08 (236.27)	.895	−458.24 (235.73)	.052	−312.73 (242.78)	.198
3+	404.78 (234.16)	.084	**−549.58** (**268.24)**	.**041**	−426.26 (285.02)	.135
Linear trend		.102		.**030**		.091
Total vBMD, mg/cm^3^						
CMD status, yes vs no	−2.35 (2.67)	.379	**−6.67** (**2.64)**	.**012**	**−6.32** (**2.68)**	.**018**
CMD burden						
0	Ref.		Ref.		Ref.	
1	−1.40 (3.20)	.662	−5.03 (2.89)	.082	−5.53 (2.92)	.058
2	−4.71 (3.43)	.170	**−8.80** (**3.33)**	.**008**	**−7.24** (**3.38)**	.**032**
3+	−1.26 (3.40)	.711	**−9.43** (**3.79)**	.**013**	**−8.20** (**3.87)**	.**034**
Linear trend		.486		.**007**		.**028**
Trabecular vBMD, mg/cm^3^						
CMD status, yes vs no	0.48 (2.48)	.847	**−4.80** (**2.27)**	.**035**	**−4.53** (**2.29)**	.**048**
CMD burden						
0	Ref.		Ref.		Ref.	
1	0.71 (2.97)	.812	−3.43 (2.49)	.168	−3.73 (2.50)	.136
2	−1.71 (3.18)	.591	**−6.49** (**2.86)**	.**024**	−5.42 (2.89)	.061
3+	2.31 (3.15)	.464	**−7.40** (**3.26)**	.**023**	**−6.58** (**3.31)**	.**047**
Linear trend		.668		.**014**		.**038**
Cortical vBMD, mg/cm^3^						
CMD status, yes vs no	**−6.92** (**2.86)**	.**016**	**−6.43** (**3.07)**	.**036**	**−6.63** (**3.12)**	.**034**
CMD burden						
0	Ref.		Ref.		Ref.	
1	−4.19 (3.43)	.222	−5.28 (3.36)	.117	−6.19 (3.40)	.069
2	**−8.22** (**3.68)**	.**026**	**−7.73** (**3.87)**	.**046**	−6.81 (3.93)	.084
3+	**−9.11** (**3.64)**	.**013**	**−8.93** (**4.40)**	.**043**	−8.57 (4.51)	.057
Linear trend		.**006**		.**033**		.058
Total mass, g						
CMD status, yes vs no	0.16 (0.13)	.215	−0.20 (0.13)	.125	−0.18 (0.13)	.183
CMD burden						
0	Ref.		Ref.		Ref.	
1	−0.03 (0.16)	.863	−0.16 (0.14)	.258	−0.16 (0.14)	.267
2	0.12 (0.17)	.469	−0.25 (0.16)	.130	−0.18 (0.17)	.272
3+	**0.44** (**0.17)**	.**008**	−0.27 (0.19)	.152	−0.25 (0.19)	.201
Linear trend		.**006**		.123		.202

Bold indicates *P* < .05.

Abbreviations: CMD, cardiometabolic disease; Ref., reference; vBMD, volumetric bone mineral density.

^*a*^Model 1 = age, race, height, weight.

^*b*^Model 2 = Model 1 + education status, family income, smoking, alcohol, physical activity, calcium intake, vitamin D intake, COPD, cancer, and antidepressants.

#### Women

The mean total strength, total vBMD, and total mass did not differ by overall CMD status in women. Subcompartment differences were observed; women with ≥1 CMD had higher trabecular vBMD (Mean [SD], 154 (39) vs 147 (37) mg/cm^3^; *P* < .001) but lower cortical vBMD (322 [47] vs 334 [43] mg/cm^3^; *P* < .001) (Table 2). In crude regression analyses, CMD status was significantly associated with higher trabecular vBMD and lower cortical vBMD (Table 4). However, after adjusting for age, race, height, and weight (Model 1) and all confounders (Model 2), there was no association between CMD status or CMD burden and total strength, vBMD, and mass (Table 4). Again, results were similar when dyslipidemia was defined based on lipid values alone compared with lipid values and self-reported medication use (data not shown).

**Table 4:** The association between cardiometabolic disease and vertebral bone strength, mineral density, and mass in women

	Crude		Model 1*^a^*		Model 2*^b^*	
	β (SE)	*P value*	β (SE)	*P value*	β (SE)	*P value*
Total strength, newtons						
CMD status, yes vs no	206.36 (119.64)	.085	−100.66 (130.94)	.442	−65.22 (135.68)	.631
CMD burden						
0	Ref.		Ref.		Ref.	
1	**355.43** (**145.64)**	.**015**	28.89 (140.39)	.837	54.23 (145.00)	.708
2	52.70 (151.84)	.729	**−382.78** (**162.24)**	.**018**	−322.69 (167.65)	.054
3+	184.22 (156.14)	.238	−112.76 (177.11)	.524	−83.49 (186.49)	.654
Linear trend		.533		.196		.292
Total vBMD, mg/cm^3^						
CMD status, yes vs no	−0.09 (2.23)	.968	−1.71 (2.40)	.476	−1.16 (2.48)	.641
CMD burden						
0	Ref.		Ref.		Ref.	
1	3.92 (2.72)	.149	0.38 (2.57)	.881	0.76 (2.65)	.775
2	−2.95 (2.83)	.298	**−6.43** (**2.97)**	.**030**	−5.30 (3.06)	.084
3+	−2.09 (2.91)	.472	−1.54 (3.24)	.636	−1.39 (3.41)	.683
Linear trend		.178		.285		.347
Trabecular vBMD, mg/cm^3^						
CMD status (yes vs no)	**7.33** (**2.02)**	**<**.**001**	−1.45 (2.11)	.491	−1.02 (2.18)	.641
CMD burden						
0	Ref.		Ref.		Ref.	
1	**6.99** (**2.46)**	.**004**	−0.63 (2.26)	.781	−0.43 (2.34)	.853
2	**6.13** (**2.56)**	.**017**	−4.30 (2.61)	.100	−3.18 (2.70)	.239
3+	**9.08** (**2.64)**	.**001**	0.75 (2.85)	.794	1.24 (3.01)	.679
Linear trend		.**001**		.907		.913
Cortical vBMD, mg/cm^3^						
CMD status (yes vs no)	**−11.51** (**2.42)**	**<**.**001**	−1.43 (2.70)	.596	−1.03 (2.79)	.711
CMD burden						
0	Ref.		Ref.		Ref.	
1	−3.04 (2.93)	.298	1.07 (2.90)	.712	1.19 (2.99)	.690
2	**−15.23** (**3.05)**	**<**.**001**	−6.24 (3.35)	.063	−5.18 (3.45)	.134
3+	**−18.26** (**3.14)**	**<**.**001**	−3.05 (3.66)	.405	−3.07 (3.84)	.425
Linear trend		**<**.**001**		.164		.204
Total mass, g						
CMD status, yes vs no	−0.08 (0.08)	.312	−0.07 (0.08)	.388	−0.06 (0.09)	.513
CMD burden						
0	Ref.		Ref.		Ref.	
1	0.07 (0.10)	.484	0.01 (0.09)	.916	0.02 (0.09)	.821
2	−0.17 (0.10)	.098	**−0.22** (**0.10)**	.**035**	−0.20 (0.11)	.063
3+	−0.17 (0.10)	.091	−0.15 (0.11)	.197	−0.13 (0.12)	.264
Linear trend		.**023**		.068		.104

Bold indicates *P* < .05.

Abbreviations: CMD, cardiometabolic disease; vBMD, volumetric bone mineral density.

^*a*^Model 1 = age, race, height, weight.

^*b*^Model 2 = Model 1 + education status, family income, smoking, alcohol, physical activity, calcium intake, vitamin D intake, chronic obstructive pulmonary disease, cancer, and antidepressants.

### Effect modification

There were many significant interactions between CMD and age in men (Table 5). CMD status was associated with lower total, trabecular, and cortical vBMD and lower total mass in younger men ≤50 years, whereas there was no significant association between CMD and the BCT measures in men >50 years of age (Fig. 2A-D). Similarly, greater CMD burden was associated with lower total (Fig. 2A) and trabecular vBMD (Fig. 2B) in younger men, whereas there was no association between CMD burden and BCT variables in older men. There were no significant interactions between CMD and race in both men and women, nor between age, menopausal status, or hormone therapy use in women (Table 5).

**Figure 2:**
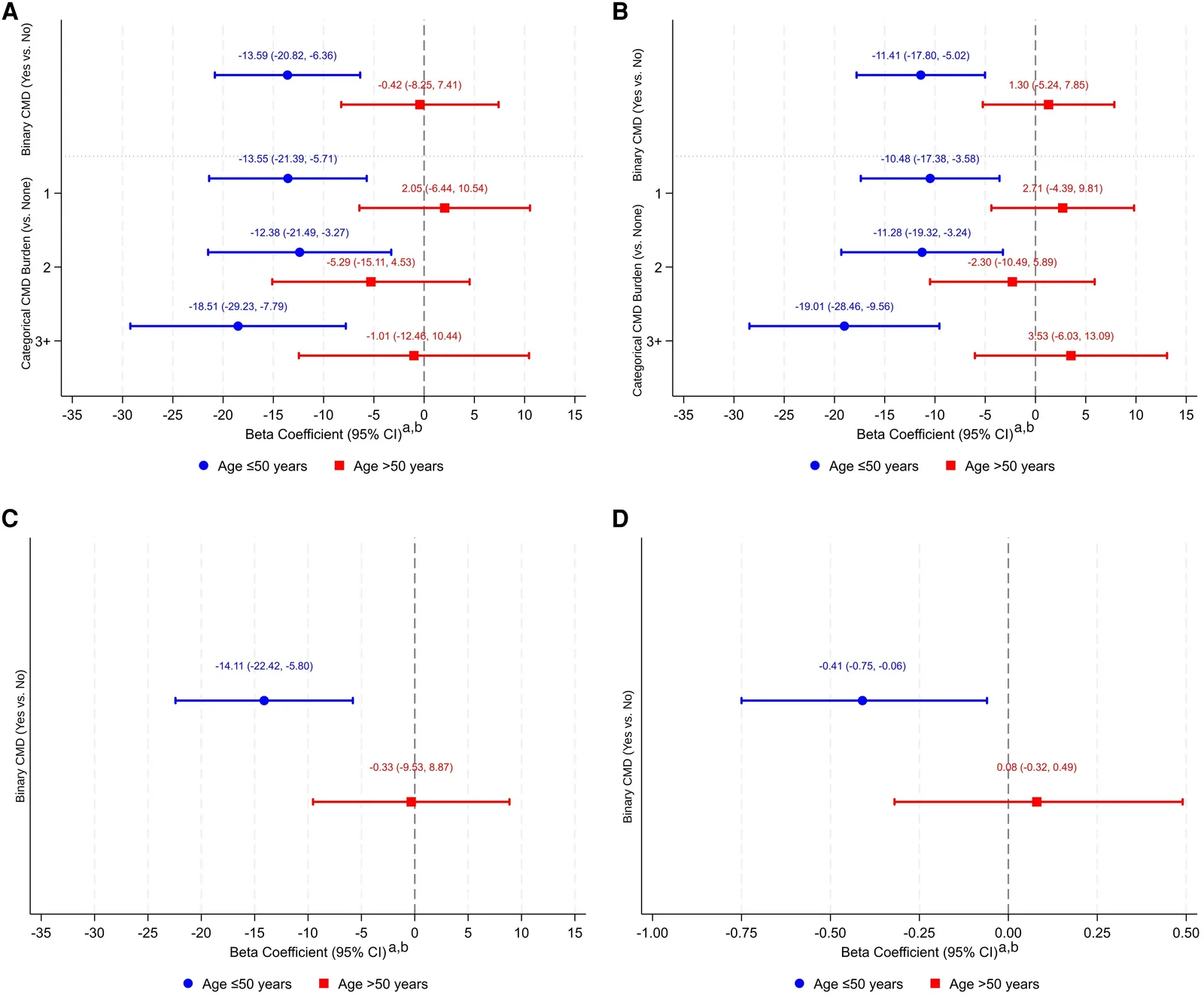
The association between cardiometabolic disease and vertebral bone mineral density stratified by age group in men. (A) Total vBMD; (B) trabecular vBMD; (C) cortical vBMD; and (D) total mass. CMD status assessed as a binary variable and categorical burden variable. Abbreviations: CMD, cardiometabolic disease; vBMD, volumetric bone mineral density. ^a^Models adjusted for race, height, weight, education status, family income, smoking, alcohol, physical activity, calcium intake, vitamin D intake, chronic obstructive pulmonary disease, cancer, and antidepressants.^b^Interaction term *P* values: Age × Total vBMD = .019; Age × Trabecular vBMD = .010; Age × Cortical vBMD = .020; Age × Total Mass = .038.

**Table 5:** Race, age, menopausal status, and hormone therapy use interaction term significance level

	Men				Women			
	Binary		Categorical		Binary		Categorical	
	*P* value*^a^*	*P* value*^b^*	*P* value*^a^*	*P* value*^b^*	*P* value*^a^*	*P* value*^b^*	*P* value*^a^*	*P* value*^b^*
Race								
Total strength, newtons	.529	.933	.255	.212	.644	.777	.984	.935
Total vBMD, mg/cm^3^	.814	.483	.338	.193	.759	.838	.941	.940
Trabecular vBMD, mg/cm^3^	.638	.187	.464	.168	.866	.713	.994	.918
Cortical vBMD, mg/cm^3^	.894	.533	.380	.256	.537	.489	.969	.923
Total mass, g	.564	.999	.189	.165	.471	.483	.673	.667
Age								
Total strength, newtons	.**034**	.058	.**044**	.114	.614	.959	.788	.867
Total vBMD, mg/cm^3^	.**011**	.**019**	.**013**	.**038**	.414	.666	.839	.955
Trabecular vBMD, mg/cm^3^	.**007**	.**010**	.**008**	.**014**	.705	.954	.962	.980
Cortical vBMD, mg/cm^3^	.**013**	.**020**	.**041**	.089	.175	.248	.426	.592
Total mass, g	.**015**	.**038**	.**041**	.116	.443	.650	.563	.736
Menopausal status								
Total strength, newtons	**—**	**—**	**—**	—	.685	.944	.719	.832
Total vBMD, mg/cm^3^	**—**	**—**	**—**	—	.193	.409	.373	.694
Trabecular vBMD, mg/cm^3^	**—**	**—**	**—**	—	.473	.818	.754	.965
Cortical vBMD, mg/cm^3^	**—**	**—**	**—**	—	.093	.185	.171	.387
Total mass, g	**—**	**—**	**—**	—	.822	.930	.610	.694
Hormone therapy use								
Total strength, newtons	**—**	**—**	**—**	**—**	.737	.833	.985	.968
Total vBMD, mg/cm^3^	**—**	**—**	**—**	**—**	.876	.785	.971	.996
Trabecular vBMD, mg/cm^3^	**—**	**—**	**—**	**—**	.435	.490	.872	.894
Cortical vBMD, mg/cm^3^	**—**	**—**	**—**	**—**	.549	.450	.900	.908
Total mass, g	**—**	**—**	**—**	**—**	.880	.933	.995	.961

Bold indicates *P* < .05.

Abbreviations: CMD, cardiometabolic disease; vBMD, volumetric bone mineral density.

^*a*^Biomechinal computed tomograpy= CMD adjusted for age, race, height, and weight.

^*b*^Biomechinal computed tomograpy = CMD adjusted for age, race, height, weight, education status, family income, smoking, alcohol, physical activity, calcium intake, vitamin D intake, chronic obstructive pulmonary disease, cancer, and antidepressants.

## Discussion

In this cohort of middle-aged, Black and White adults, we found that having at least 1 of the CMDs of interest was associated with lower total and cortical vertebral vBMD in men, whereas there was no relationship between any CMD and any BCT variable in women. Similarly, greater CMD burden was associated with lower vBMD in men, with the strongest associations seen in men with ≥3 CMD conditions. The inverse relationship between CMD (status and burden) and vBMD was statistically significant in younger (≤50 years) compared with no association in older (>50 years) men. Race did not modify the relationship between CMD and bone strength in either men or women. Age, menopausal status, and hormone therapy use did not modify the association between CMD and vertebral bone strength, density, or mass in women.

To our knowledge, this is the first study evaluating the association between CMD and BCT bone properties other than DXA-based BMD. Previous studies evaluating the relationship of CMD expressed as metabolic syndrome or as the individual conditions used trabecular bone score (TBS) or high-resolution peripheral quantitative CT (HRpQCT) to measure bone structural properties. TBS quantifies gray-scale variations in the DXA image reflecting bone texture, providing an indirect assessment of bone microarchitecture overall (65-67). Earlier studies showed lower TBS (ie, worse microarchitecture) in those with metabolic syndrome or T2DM despite higher DXA BMD (68). However, more recent studies showed that this association was driven by soft tissue and not directly attributed to T2DM (33, 69-71). HRpQCT directly measures microarchitecture in trabecular and cortical bone at the radius and/or tibia instead of central sites (femoral neck and lumbar spine) used in DXA. Most of the HRpQCT studies are limited to T2DM, which have consistently reported no difference or sometimes greater trabecular BMD and microarchitecture (eg, trabecular number and thickness) in those with compared to those without T2DM (33). The relationship between T2DM and cortical bone has been mixed, with some showing greater cortical porosity, especially in those with microvascular disease (72, 73), whereas others reported no significant difference in cortical parameters at the tibia (74). Our findings in men only confirm the greater body of HRpQCT evidence showing an adverse relationship with cortical bone. The differences in findings could be explained by our composite CMD variable instead of focusing on T2DM specifically.

There is limited information in population-based studies regarding bone strength and the other conditions included in our CMD exposure (75, 76). One small study on dyslipidemia and bone strength evaluated the relationship between atherogenic index of plasma (a ratio of triglycerides to HDL-C) and TBS and reported higher atherogenic index of plasma was significantly associated with 60% higher odds of low TBS (< 1.230), independent of age, body mass index, smoking, previous fracture, kidney function, and lumbar osteoporosis assessed via DXA in postmenopausal women (77). On the cardiovascular disease side, a previous study in older adults from the United Kingdom reported that ischemic heart disease was associated with lower cortical vBMD and higher cortical porosity at the distal radius, and similar to our study, this association between ischemic heart disease and cortical vBMD was only significant in men (78).

Numerous studies have evaluated the possible mechanisms through which CMD is associated with bone, including dyslipidemia impacting osteocyte function and increasing bone resorption (79); hyperglycemia and visceral obesity impacting osteoblast function, disrupting bone formation (80); advanced glycation end-products reducing bone elasticity (81); and chronic inflammation associated with most of these conditions enhancing osteoclast activity (80, 82, 83). This lower formation and higher resorption can lead to low bone strength, density, and mass. These mechanisms of action ideally should be the same in men and women, but we only found a significant association in men. The men in our study had a higher prevalence of dyslipidemia, so it is possible that factors related to dyslipidemia are driving these sex differences. Similarly, the women had a higher prevalence of abdominal obesity. Adipose tissue and estrogen have a complex relationship, with both positive and negative effects on bone (84), so a deeper exploration into these pathways could further elucidate information on the relationship between CMD and vertebral strength properties in women. Lastly, more research is needed to explore the role sex hormones play in the relationship between CMD and bone health.

This study has many strengths, such as the inclusion of novel vertebral bone measures in Black and White men and women from a community-based cohort study. Given CARDIA's standardized protocols and extensive data collection, our CMD exposure is highly valid. Likewise, the CARDIA imaging protocols yielded high-quality CT scans, minimizing missingness in the BCT data. The use of CT-based BMD measures reduces the high variability and limited reproducibility of BMD measurements associated with fat layering seen in DXA (85).

Despite these strengths, our study is not without limitations, including its cross-sectional design and limited age range of participants. The clinical definitions for the CMDs have changed throughout the course of the CARDIA study. We used the most current definitions to be consistent with today's clinical practice, even if definitions were different at year 25 (2010-2011). CARDIA did not measure biomarkers specific to type 1 diabetes mellitus, which affects bone differently than T2DM. Misclassification of diabetes type is possible, but T2DM represents 90% to 95% of people with DM (86), so we anticipated minimal misclassification. Our abdominal obesity definition was based on waist circumference, which is correlated with body weight. However, by retaining weight as a covariate, we were able to explicitly evaluate abdominal obesity and not general weight-based obesity. We also did not adjust for antiobesity medications. The first-generation antiobesity medications (eg, orlistat and phentermine) available in the study period (2010-2011) did not advance expected weight loss much above the 3% to 8% observed from the intensive lifestyle interventions in the Diabetes Prevention Program (87). There are also no data to show that these older medications are associated with improved glucose control, insulin resistance, or reduced cardiovascular disease, thus limiting the effect of these medications on bone. Our CMD exposure was a composite of 4 variables, of which 3 had multiple avenues in which to meet the definition, leading to heterogeneity within each condition. Our future analyses will parse out this heterogeneity, along with identifying which condition or group of conditions are driving the associations observed.

Outside of height and weight, all potential confounders were self-reported. Self-reported data are prone to information bias, which could lead to misclassification and increase the type 1 error rate. Our nutrition data were carried forward from year 20. As previously mentioned, dietary habits in adults are relatively habitual. Those newly diagnosed with 1 of the CMDs between years 20 and 25 may have undergone dietary changes. Dietary changes for cardiovascular and metabolic health involve reducing refined carbohydrates and increasing fruit and vegetable consumption, so there could have been an increase in calcium and vitamin D intake, which would have a benefit on bone. Given the potential for incorrect nutrition data, our observed findings could be an over- or underestimate of the true relationship between CMD and bone outcomes. Our study included 5 outcomes and 2 exposures, with additional effect modification analyses, potentially warranting adjustment for multiple comparisons to minimize the family-wise error rate (88). However, our 5 outcomes were not all independent of each other. Vertebral strength is highly correlated with density and moderately associated with size. Likewise, total vBMD is essentially the combination of trabecular and cortical vBMD. Given the correlations between our outcomes, we did not formally adjust for multiple comparisons. Lastly, though we evaluated and adjusted for many potential confounders, as with any epidemiologic study, residual confounding could remain.

## Conclusion

In this diverse cohort of US adults, CMD was significantly associated with lower overall and subcompartment vertebral vBMD in men only. Our findings can be used by endocrinologists and other clinicians managing patients with CMD, particularly in younger men, to support monitoring bone health (eg, bone turnover markers, CT imaging) in this population. This cross-sectional study served as the foundation for a future longitudinal study evaluating how CMD is associated with changes in bone health and vertebral fractures. Future analyses evaluating the causal mechanisms to our observed findings could lead to the addition of CMD or related biomarkers in fracture risk prediction models.

## Data Availability

CARDIA data are available upon reasonable request from the CARDIA Coordinating Center. CARDIA investigators are eager to collaborate with investigators interested in using CARDIA data. Please see the CARDIA website (https://sites.uab.edu/cardia/) for publication policies and for a list of CARDIA Representatives. CARDIA data are also publicly available on the NIH-supported BioLINCC and dbGaP platforms.
